# Differences between the European Union and United States of America in Drug Regulatory Affairs Affect Global Patient Safety Standards and Public Health Awareness: The Case of Deferasirox and Other Iron Chelating Drugs

**DOI:** 10.3390/medicines8070036

**Published:** 2021-07-07

**Authors:** George J. Kontoghiorghes

**Affiliations:** Postgraduate Research Institute of Science, Technology, Environment and Medicine, Limassol 3021, Cyprus; kontoghiorghes.g.j@pri.ac.cy

**Keywords:** patented drugs, deferasirox, deferiprone, deferoxamine, ethics, regulatory affairs, patient safety, drug efficacy, orphan drugs, marketing, public health

## Abstract

Regulatory policies on drugs have a major impact on patient safety and survival. Some pharmaceutical companies employ all possible methods to achieve maximum sales in relation to the monopoly of their patented drugs, leading sometimes to irregularities and illegal activities. Misinformation on the orphan drug deferasirox has reached the stage of criminal investigations and fines exceeding USD 100 million. Additional lawsuits of USD 3.5 billion for damages and civil fines were also filed by the FBI of the USA involving deferasirox and mycophenolic acid, which were later settled with an additional fine of USD 390 million. Furthermore, a USD 345 million fine was also settled for bribes and other illegal overseas operations including an EU country. However, no similar fines for illegal practises or regulatory control violations have been issued in the EU. Misconceptions and a lack of clear guidelines for the use of deferasirox in comparison to deferiprone and deferoxamine appear to reduce the effective treatment prospects and to increase the toxicity risks for thalassaemia and other iron loaded patients. Similar issues have been raised for the activities of other pharmaceutical companies promoting the use of new patented versus generic drugs. Treatments for different categories of patients using new patented drugs are mostly market driven with no clear safeguards or guidelines for risk/benefit assessment indications or for individualised effective and safe optimum therapies. There is a need for the establishment of an international organisation, which can monitor and assess the risk/benefit assessment and marketing of drugs in the EU and globally for the benefit of patients. The pivotal role of the regulatory drug authorities and the prescribing physicians for identifying individualised optimum therapies is essential for improving the survival and safety of millions of patients worldwide.

## 1. Introduction

Pharmaceuticals are a major source of income mainly for developed countries. Annual drug sales by the top ten world pharmaceutical companies, which are mainly involved in the sale of new patented drugs are estimated to exceed USD 0.5 trillion [1]. There is fierce competition worldwide in the supply and sale of medicinal drugs, including generic and new patented drugs.

In many cases, drug selection for the treatment of a specific condition is not clearly defined due to gaps or loopholes in regulatory, marketing and other policies. This can affect the safety and long-term survival of different categories of patients [1,2,3,4,5,6,7]. In some cases, these issues arise from methods used by pharmaceutical companies to achieve maximum income from the manufacturing and supply of the drugs for which they have been granted exclusive worldwide patent protection and monopoly on sales. Patients can end up with suboptimal drug therapy in terms of toxicity, efficacy and cost, mainly as a result of the interplay between the activities and policies of the government and pharmaceutical industry [1,2,3,4,5,6,7,8,9,10].

**Figure 1:**
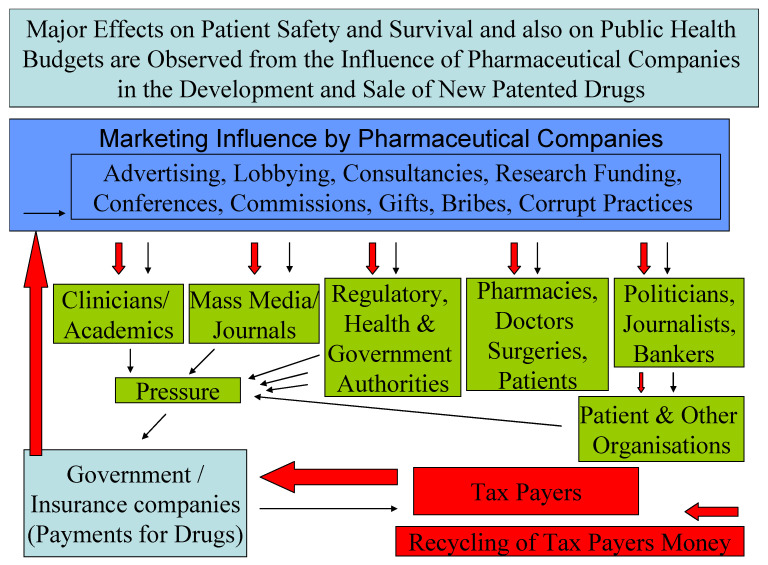
Ethical issues arising from the influence of pharmaceutical companies regarding new patented drugs. A flow chart describing the marketing influence of pharmaceutical companies on various sectors and organisations in relation to new patented drugs and also its effect on patient safety and public health budgets.

There is potential for ethical and indeed legal conflicts in the promotion and distribution of new patented drugs, as evidenced by past unlawful activity undertaken by physicians, academics, drug regulatory authorities and patient organisations [1,2,3,4,5,6,7]. Nondisclosure agreements between academics or institutions and pharmaceutical companies can lead to biased or inaccurate reporting of clinical trial results [1,2,3,4,5,6,7]. While it is likely these pitfalls will always be present in some form or another, it appears that in the European market, consumers and in particular patients would benefit from a stronger central framework for assessing wrongdoing by pharmaceutical companies and delivering punitive measures when due (Figure 1).

## 2. Ethics and the Pharmaceutical Industry

While great strides have been made in the regulation of the pharmaceutical industry, from lead drug identification to post-marketing surveillance, there are many opportunities for the drug development process to be subverted from patient benefit being the primary goal. Investment in clinical research comes almost exclusively from industry, which is required to turn a profit, thus modifying publication and prescribing processes which can be commercially motivated [1,11,12,13].

A survey in the USA indicated a bleak picture for the state of contracting between the academic institutions and pharmaceutical industry, where very few research centres included standard language in their contracts that guaranteed, for example, the investigators’ access to the primary data from the entire study [14].

Cases of misconduct involving physicians and the pharmaceutical industry, in relation to financial conflicts, clinical trial findings, and drug prescription patterns, have been widely reported [2,15,16,17,18,19]. In Greece, physicians in charge of enrolling thalassaemia patients to convert from generic drugs to a much more expensive new patented drug in a post-marketing surveillance study were paid EUR 5000 per patient [1,7,20]. Cases involving bribery of clinicians have also reached the courts. In one German case, ‘donations’ of up to EUR 10,000 had to be allowed for the promotion of medical products by private clinicians, due to the sheer quantity of instances. Most of the recipient physicians were also working for the German National Health System [21].

There is therefore a thin line between ethics and commercial activity by the pharmaceutical industry, academic institutions, hospitals, drug regulatory authorities and other similar bodies. In this context, self-regulation by the industry has proven in many cases inadequate for providing optimal individualised therapies for patients. For example, the development of drug combination therapies which may provide more effective treatments in comparison to monotherapies and also the development of drug antidotes for minimising toxic side effects are not encouraged by the industry for financial reasons [1,20]. With profit taking the front seat in decisions on drug development, it is perhaps not surprising that unethical or seemingly malicious activities are undertaken at the expense of patients.

Misinformation on drug toxicity and efficacy, risk/benefit assessment, drug pricing and the therapeutic index of new patented drugs in comparison to generic drugs can all have direct effects on patient safety and long-term survival and also government health budgets [13,22,23]. Different rates of survival of thalassaemia patients receiving iron chelation therapy were observed as a result of the timing of approval and use of deferiprone (L1) in India in 1995, the EU in 1999 and the USA in 2011 [1]. Major differences in the sale price of the orphan chelating drugs deferasirox (DFRA), deferiprone (L1) and deferoxamine (DF) has affected their availability to patients in developing countries where health facilities and finances are scarce. In many countries, local production of deferiprone and deferasirox has mostly overcome the problems of high prices of imported formulations [1].

Further cost reduction was anticipated with the expiration of the deferasirox patent in 2017. However, new patented formulations of deferasirox with the same active ingredient and questionable policies excluding generic companies involved in the production is costing the public for example in Europe about 64,700 euros per a 75 kg adult patient using a dose of 40 mg/kg/day. The cost of production of generic deferasirox is estimated to be about 0.1% of the sale price. This patent extension monopoly policy is not unique for deferasirox but is observed in most other drug cases where the patent has expired and is mostly benefiting big pharmaceutical companies in wealthy countries at the great expense of public health funds and also patients in developing countries.

Marketing teams within each pharmaceutical company employ sophisticated strategies to promote sales (Figure 1) [1,2,3,4,5]. Settlements reached prior to court rulings often ensure that companies need not admit blame when such activities extend across ethical and legal lines [1,3]. These strategies are many and varied and have extended into the development of iron chelating drugs [1,24,25,26]. One example that reached the courts and global media was the claim by a clinician that she was prohibited by the sponsoring company from publishing clinical data related to the chelating drug deferiprone suggesting that it was hepatotoxic and ineffective [25]. The subject was debated by many parties, including the clinician’s research collaborators, the sponsoring company, patients’ organisations and the institution where the work was performed [26,27,28,29]. This was accompanied by outcry for academic freedom by eminent academics and the organisation of ethical symposia, court battles in the EU and elsewhere, a ban of the drug in the USA and Canada amongst other developments [27,28,29].

Eventually deferiprone was confirmed by many other clinical studies not only to be non-toxic to the liver but to be life saving for thalassaemia patients [1,29,30,31,32]. Furthermore, it appears that the case against deferiprone was organised and sponsored by the manufacturers of deferasirox in order to delay the further development and marketing of deferiprone until deferasirox received regulatory approval [1,24]. In addition, several academics who spoke against the introduction of deferiprone in thalassaemia patients were consultants and received sponsorship from the manufacturers of deferasirox [1,28].

Clearly, government intervention and stricter regulation are necessary for safeguarding patients’ and society’s interests. Some encouraging actions and campaigns have been initiated by international and national bodies such as the WHO and NICE in the United Kingdom to overcome problems related to optimal drug selection, patient safety and drug overpricing [13,22,23]. However, even in these cases, such actions proved ineffective or misleading, e.g., on chelating drugs or vaccines for the A H1N1 influenza virus since the decision makers were either themselves consultants or associated with pharmaceutical companies [1,9,33].

## 3. A Brief Update on the Clinical Use of Iron Chelating Drugs

Reports from results of clinical trials with chelating agents have in general yielded conflicting results. However, a post-marketing monitoring assessment of deferasirox conducted by the EMA observed an 11.7% mortality rate, which is of course clear cause for concern [1,6,34,35,36]. By comparison, the mortality rate for patients prescribed deferasirox’s generic competitor drugs, deferoxamine and deferiprone, has been estimated to be less than 0.1% [37]. Furthermore, these generic competitor chelating drugs, especially deferiprone, appear to be more effective in removing excess cardiac iron and preventing congestive cardiac failure, which is a primary cause of mortality in thalassaemia patients [1,6,7,30,31,32,38]. The weight of evidence suggests individualised protocols using deferiprone alone or in combination with deferoxamine can normalise iron stores and improve long-term survival rates in thalassaemia and other iron chelation patients, with a low incidence of toxic side effects [31,37,39,40,41]. A summary on the mode of action and the clinical effects of the three iron chelating drugs is shown in Table 1.

Contrary to clinical findings the manufacturer of deferasirox initiated a strong worldwide marketing campaign and distributed information suggesting that the drug was equally or more effective in removing iron from iron loaded patients in comparison to deferoxamine and also safe. However, in addition to the overt safety concerns, deferasirox was not effective in the removal of excess cardiac iron or in normalising the iron stores in iron loaded patients [38].

These marketing activities appear to have impacted prescribing patterns. For example, in Cyprus, where marketing activity was monitored due to the high cost of deferasirox and the approval for its use relied upon an independent physician committee risk/benefit assessment, only 15–20% of patients on chelating drugs are using deferasirox [1,6,31,34,37]. Similar policy in the use of deferasirox as a second line treatment was suggested by the Italian Society of Haematology [42]. In contrast, in other European countries and also the USA, where the “free choice” policy on drug selection by physicians applies, a higher proportion of iron-loaded patients are using deferasirox. It has also been established that several physicians and other healthcare professionals chose not to mention to patients the possible fatal side-effects of the drug, including renal, hepatic, bone marrow and haemorrhagic episodes, as well as many other serious toxic side effects which are included in the drug label as shown in Table 2 [1,6,34].

Deferasirox is primarily recommended for patients with insufficient response to or low tolerability for deferiprone and or deferoxamine and or deferiprone/deferoxamine combination treatment. It is widely used in many countries, but reports of low efficacy and high toxicity are frequent [1,31,35,36,37,38,43,44]. Attempts at increasing the maximum suggested dose of deferasirox from 30 to 40 mg/kg/day and expanding the indication to include non-transfusion-dependent thalassemia, presumably to match the efficacy profiles of the competitor chelating drugs, seem risky given the serious and fatal toxicities observed in animal and human studies (Table 2) [6,9,34,43]. Some studies suggest combining deferasirox with either deferoxamine or deferiprone can improve the therapeutic profile, but further investigations, including comparison with the deferiprone/deferoxamine combination, are needed to confirm such findings [45,46].

The level of safety of each of the chelating drugs can be deduced from recent clinical studies involving non-iron loaded categories of patients. Major evidence regarding the safety of deferiprone in particular has been obtained from its identification as one of the leading pharmaceuticals for the treatment of neurodegenerative diseases including Friedreich’s Ataxia and pantothenate kinase-associated neurodegeneration (Table 1) [32,47,48,49,50,51,52].

## 4. The USA Response to Misinformation on the Safety of Deferasirox

Deferasirox was developed and approved under the orphan drug pathway in the USA and EU, providing “relaxed toxicity screening” and extended patent protection in these markets. The commercial influence and drug marketing tactics used to support this patented drug, in comparison to other generic iron chelating drugs, have been previously described [1,9].

In the USA, criminal proceedings have been brought against the manufacturer of deferasirox for alleged misinformation about the safety of the drug. Fines have been imposed against the manufacturer, with one civil fraud lawsuit reaching settlement of USD 60 million for understating life-threatening toxicities and another settlement of USD 45 million being reached over false claim allegations being submitted to federal health care programmes [53,54]. Furthermore, in 2015, the FBI of the USA Government filed a lawsuit of USD 3.5 billion for damages and civil fines involving deferasirox and mycophenolic acid [53,54,55]. The case of deferasirox and mycophenolic acid was settled with a fine reaching USD 390 million [56].

Recently in 2020, a total of USD 345 million fine was settled by the USA Government department of justice in cases related to the manufacturer of deferasirox, to resolve criminal and civil charges for bribes of doctors, hospitals, etc., and other illegal overseas operations which took place in South Korea, Vietnam and Greece, an EU member state [57].

In Greece, the illegal operations regarding the manufacturers of deferasirox has reached major publicity and were termed “the scandal of the century” of modern Greece. Ten top politicians including an ex-prime minister, other ministers, a central banker and a member of the EU council were accused of taking bribes [58]. The present Greek government is also seeking compensation from the manufacturers of deferasirox over the bribery revelations involving Greece, which were identified by the USA Government department of justice [57,59].

There have been no similar fines issued in the EU courts for illegal practices or regulatory control violations as those issued in the USA courts. Lawsuits of this nature are not unprecedented in the USA. Previous interventions by the USA authorities in other companies have included fines to settle civil and criminal investigations in relation to sales and practices of other drugs. In two cases, a total of USD 2.3 billion and USD 3.0 billion in fines were respectively imposed for similar irregular activities [1,3].

In contrast to the intervention of the USA regulatory authorities in relation to deferasirox toxicity misinformation, no similar cases have been initiated or steps taken by the drug regulatory authorities or other appropriate government bodies in the EU [53,54,55,60]. Compared to the USA authorities, the EU drug regulatory authorities have hesitated to act or intervene and have not imposed any fines on the deferasirox manufacturer. In this and other cases, the intervention of the EU drug regulatory authorities is slow and of a much lower level compared to that of the regulatory drug authorities in the USA [1,2,3,4,53,54,55,56,57,60].

## 5. Suggestions and Opportunities for an Improved Drug Regulatory Framework in the EU

It appears that in general, the controls against unethical and illegal behaviour regarding medicinal drugs are tighter and penalties are higher in the USA, in comparison to other countries including member states of the EU [5,15,19,20,60]. It also appears that the unified and centralized framework adopted in the USA is more efficient and effective in identifying and preventing such cases, whereas in the EU there is no similar centralized system or legal framework. Individual member states are expected to act on their own will and apply their relevant national laws [4,10,15,20,21,22,59,60]. In this context, legal and other initiatives need to be pursued in the EU in order to close loopholes and apply measures in the pharmaceutical industry that can improve drug safety standards and treatment outcomes for patients and also improve public health in general not only in the EU but also worldwide. Some of the issues related to deferasirox and other drugs are outlined below.

### 5.1. Close the “New Formulation” Loophole

The “new formulation” concept which may involve an active pharmaceutical ingredient formulated at different doses or using different additives has been exploited financially by the pharmaceutical industry for many years causing a major impact to public health costs and drug availability to patients [7,8,61,62]. In this context, a new formulation of deferasirox has been patented and approved and recently introduced in an attempt to maintain the huge profit margins and monopoly of the proprietor company, even though such developments will not bring significant clinical improvements or benefits and also will not be in the best interest of patients and of public health in general [63]. Secret dealings among pharmaceutical companies are common and are likely to prevent the production of cheaper versions of generic deferasirox, similar to many other cases including those of deferiprone, deferoxamine and other generic drugs [1]. Overall, a higher burden of evidence of clinically significant improvements will be required when developing new drug formulations while at the same time ensuring that the generic version is widely available and produced by generic companies not associated to the previous patent holder company.

### 5.2. Improve Ability to Identify “False” Patient Subgroup

Patent applications have also been filed for the use of deferasirox for the treatment of the so called “non-transfusion-dependent-thalassaemias” despite that this thalassaemia intermedia group of patients has existed and been treated effectively for the past 50 years with deferiprone and deferoxamine [37,64]. There is no difference in the use of chelating drugs for thalassaemia major, intermedia, “non-transfusion-dependent-thalassaemia” and other similar categories of patients with equivalent levels of iron overload [1,37,64]. The regulatory approval of deferasirox has no advantages for this category of patients and such developments are not in the public health interest. It appears that the safety and survival of most of the deferasirox treated patients will be negatively affected in comparison to patients with “non-transfusion-dependent-thalassaemias” treated with deferoxamine and deferiprone [1,27,31,37,39,40,41,64].

Similar low efficacy and high toxicity findings, as well as high risk/low benefit prospects of treatment have been identified in relation to the use of deferasirox in other iron loaded patient subgroups such as myelodysplasia and myelofibrosis, sickle cell anaemia, post-allogeneic hematopoietic stem cell transplantation, idiopathic haemochromatosis and also in other non-iron loaded subgroups including cancer and other categories (Table 3) [6,24,34,43,65,66,67,68,69,70,71,72,73,74,75].

### 5.3. Aim of Therapy and Risk/Benefit Assessment of Available Drugs in the Era of Personalised Medicine

In considering the treatment of diseases in general, the aim of any drug therapy and the therapeutic targets to be achieved should be clearly specified. There is therefore a great need for implementing objective therapeutic targets for the treatment of diseases without relying on the subjective selection of drugs by physicians, which may be influenced by drug marketing procedures. If the therapeutic target can be achieved by all available drugs, the need for such implementation is not necessary despite that in most cases public health drug authorities may insist on the cheaper options.

Full information on all aspects of available therapeutic options for each disease can facilitate the selection of the best possible treatment for patients. Most importantly, all the information related to all the findings related to candidate/selected drugs, from the chemical synthesis to clinical results in post marketing surveillance, should become widely available.

Furthermore, bearing in mind that each patient’s response to any drug is different and depends on the level of absorption, distribution, metabolism, elimination and toxicity (ADMET) and also on other factors such as pharmacokinetics, tolerance, nutrition, drug interactions and organ function, there is a need to adequately prove that a new drug is better than comparators while also accounting for subgroup variations in each case [27,75,76,77]. In this context, personalised medicine can be used to optimise therapy for subgroups of iron loaded and other groups of patients [39,41,78,79].

### 5.4. Improve Transparency

Full transparency on all aspects of drug development including post-marketing surveillance will result in better and safer treatments for patients. Unbiased reporting and disclosure of the results of clinical and non-clinical studies, including toxic side effects, competition studies with generic drugs at optimal doses, agreements between academics/academic institutions with the pharmaceutical companies, and determination of drug prices are essential for securing patient safety and also in the public health interest [1,8,9].

Steps should also be taken to improve transparency regarding medical journals, which are the major contributors in the dissemination of basic and clinical science information guiding physicians in the selection of therapeutics. Instead of independent assessments, most of the clinical trial results on the effects of new therapeutics are authored by academics founded or sponsored by pharmaceutical companies [17,80,81,82]. Similarly, many members of editorial boards and referees of medical journals are not only affiliated to academic institutions but also to pharmaceutical companies. In addition, most publications related to new patented drugs are usually biased in relation to efficacy and safety and are controlled by medical writers affiliated to the pharmaceutical companies [1,24]. Such information is recycled in the medical community with repeated publications and citations of only positive results, which are attributed to only authors collaborating with the pharmaceutical companies [24]. The independent assessment of articles regarding new drugs by academics not affiliated to pharmaceutical companies and the publication of pharmaceutical companies’ sponsored articles as advertisements could improve transparency and increase the prospects of unbiased reporting regarding the safety and efficacy of new drugs.

The relationship of journals, including elite journals such as the New England Journal of Medicine and the Lancet with the pharmaceutical industry should also be investigated. In both cases, the publication of misleading information regarding chelating drugs associated with the pharmaceutical industry, as well as the publication of false information related to drug development and plagiarism, appears to point to “non-disclosure” sponsorship of the journals by pharmaceutical companies [24,83,84,85,86]. Similarly, the promotion of chelating drugs on behalf of pharmaceutical companies in journals also appears to be undertaken by physicians who are public health employees in different countries and who are also acting as consultants and funded by pharmaceutical companies [87]. Transparency and access to journal and all other data related to health is essential for safeguarding and improving patients’ survival and safety [88].

### 5.5. Improve On-Going Safety Monitoring, Especially for Orphan Drug Indications

The monitoring of a drug’s efficacy and toxicity at all phases of development is essential and necessary for the full evaluation of drugs [1,6,9]. Even at the post-marketing surveillance stage, improved monitoring methods can be introduced to detect any rare or long-term adverse effects in a larger patient population, which was not available during the previous clinical trial phases. It should be noted that many drugs have been withdrawn or their use restricted due to toxicity at this stage, e.g., rofecoxib [89].

Once approved, a greater burden must apply to orphan drugs due to the more relaxed regulations for approval and the state funded financial incentives involved in development. In this context, sufficient drug information should become available by physicians to patients with emphasis on the toxic side effects and also the possibility of other safer and more effective drug treatment options.

### 5.6. Increase Involvement of Academic and Healthcare Institutions in Drug Development

Speedy and safe development of new drugs can be achieved with major involvement of independent academic sectors of public universities, hospitals and other specialist institutions at all stages, including the assessment of clinical results. In particular, initiatives in drug design and development including clinical trials should be encouraged in a model similar to what has been used for the development of deferiprone, based on academic initiatives, patients’ needs and patient organisation participation [1,86]. This route of drug development, which lasted less than 10 years, was estimated to be about 100 times less expensive compared to that of the private sector [1,86]. It should be noted that shortcuts to all regulatory routes can be implemented, and pharmaceuticals can be supplied without a license in cases of emergency including the present COVID-19 pandemic, thus questioning the present system of long-term drug development [33].

Academic and healthcare institutions could also be involved in many other research activities which are not funded by the pharmaceutical industry but could increase patient safety standards and improve drug outcome treatments. Such activities could involve the identification of the mechanisms of drug toxicity including drug interactions, the design of drug antidotes and toxicity preventative measures, the introduction of drug combination therapies, the design of safer and more effective personalised drug protocols and many others [79,90].

### 5.7. Stricter Monitoring and Controls on Marketing Activity

Medicinal drugs are mainly considered to be a commercial commodity and their use to be market driven. The lobbying activities of pharmaceutical companies in the promotion of new patented drugs extends across community and government sectors, including physicians, patients, government regulatory authorities, EU authorities, academic institutions and academic journals, sometimes with undesirable effects (Figure 1) [1,2,3,4,5,6,7,80,81,82,91]. In this context, the introduction of stricter legal measures and regulatory controls is essential for limiting all irregularities and illegal issues which can directly or indirectly affect patient safety and treatment outcomes. Similarly, the determination of drug prices and cost effectiveness of both new patented and generic drugs, also affect patient treatments in developed and developing countries as well as new drug development and applications [70,92,93,94,95,96,97,98,99,100]. Excluding the expenditure related to drug marketing from the total cost of drug development may reduce drug prices, government health budgets, irregular or illegal activities, as well as the safeguarding of patients’ interests for safer and more effective drug treatments [1,96].

### 5.8. Monitoring and Tackling Unethical and Illegal Activity by the Pharmaceutical Industry in the EU

Corruption in global health including the area of pharmaceuticals is an open secret and most health authorities including those in the EU turn a blind eye, despite that patient lives are affected and may be at risk [1,101,102].

In relation to pharmaceuticals, there are many grey areas and loopholes between the pharmaceutical industry and individual EU state laws in securing optimal treatments for patients and also for achieving maximum safety. Patients’ lives are at risk from misinformation or insufficient information on drug toxicity and also from false risk/benefit drug assessments. In many cases, the suggestion that a new drug is better than an old one despite the lack of sufficient evidence and especially providing inaccurate or misleading information on safety is not only unethical but also criminal. There are very thin lines between marketing practices by the pharmaceutical industry and the safeguarding of patients’ rights for safer treatments, which appears not to be specified or clarified sufficiently enough in the EU state laws in comparison to USA.

New monitoring and legal structures should be implemented in the EU, since self-regulation on marketing and safety issues in the pharmaceutical industry appear at present to be insufficient and ineffective. The main aim of these structures will be to tackle related problems for the achievement of higher safety standards and optimal therapies for patients in the EU. The present system adopted for monitoring and tackling these issues by individual member states of the EU appears not to be effective at least in comparison to the USA system. It seems that developing responsibility of a central EU court in conjunction with the EMA and Europol, to monitor unethical/illegal activity in the pharmaceutical industry and to bring legal action against the relevant companies and their associates, is more appropriate and possibly more effective than relying on individual member states.

## 6. Future Prospects for Minimizing Differences in the EU and USA in the Interest of Public Health

The primary purpose of regulatory procedures for medicinal drugs worldwide is to safeguard public health, and this can generally be achieved, provided regulatory drug authorities can ensure that pharmaceutical companies comply with the necessary regulations.

The largest volume of medicinal drug investigations and approvals worldwide are carried out in the USA and in the EU. Similarly, the top ten pharmaceutical companies producing new patented drugs are also based in these countries [1]. Despite drug approval processes in the EU and USA being the most demanding in the world, there are many insufficiencies, loopholes and differences in both systems that can have an effect on patient treatment and public health not only in the EU and USA but also worldwide [102].

The many differences and complexities in the regulatory procedures for drug development and clinical use between the EU and USA stem mostly from the different philosophies and approaches following the formation of the FDA and EMA, respectively. In general, whilst the development of the FDA was based on the premise of serving as a centralized consumer protection agency in the USA, the purpose of the development of the EMA was to harmonize inter-state commercial interests and legislation regulations among the 27 member states of the EU [103].

The drug regulatory procedure differences between the EU and USA include amongst others the areas of drug post-marketing surveillance, emergency medicines and orphan drugs such as deferasirox, deferiprone and deferoxamine [1,104]. It appears that in general, the FDA is primarily focused on safety concerns regarding drugs for the protection of consumers/patients at the cost of commercial enterprise, whereas the systems in European countries and the EMA are primarily concerned with preserving commercial interests and less with patient safety [103]. These differences can be highlighted by law enforcement and penalty levels. In this context, lawsuits against pharmaceutical companies in the USA are very common in regard to numerous violations including financial conflicts, clinical trial findings and reporting, drug prescription recommendations and advertisements as well as bribes. In contrast to the stringent system in the USA, in European countries, the mixture of government and private processes regarding drug regulatory affairs monitoring and post-market surveillance results in a more relaxed and less efficient system. Accordingly, heavier fines and out of court settlements for misconduct are more frequently observed in the USA than the EU.

Despite their differences and taking into consideration the benefits related to patient safety, both the EMA and FDA are discussing programmes of collaboration and exchange of information to strengthen efforts on drug safety in both their regions and worldwide [105]. These processes have been accelerated as a result of the COVID-19 pandemic, which has highlighted the need for one world/one health policy for the benefit of humanity [101,106].

## 7. Conclusions

Chelating and other medicinal drugs are generally considered a commercial commodity with their use market driven, thus decreasing the prospects for optimal personalised therapy for patients with thalassaemia and other iron loaded conditions as well as other non-iron loaded diseases.

It appears that differences between the USA and EU regulatory monitoring drug procedures and controls in relation to deferasirox affect the safety and survival prospects of thalassaemia and other patients. In contrast deferiprone and its combination with deferoxamine increase the survival of thalassaemia patients by reducing cardiac mortality and by eliminating excess toxic iron to normal physiological levels.

The sincerity and hard efforts of all those involved in drug development and the introduction of new drugs including the vast majority of pharmaceutical companies, physicians, regulatory authorities, academics and others is widely recognised. From society’s and patients’ perspective, ethical issues related to improvements on patient safety and survival, including the introduction of better drugs as well as conducting life-saving pre-clinical and clinical research and clinical trials properly and quickly, is a moral imperative. In this context, the maximum cooperation and collaboration of all those involved in drug design and development is essential and necessary for patients and society in general.

However, in reality, ethical issues are of secondary importance to the pharmaceutical industry where financial success is the main aim. The existence of pharmaceutical companies relies on marketing, income and sales ahead of patients’ treatment outcomes or safety. The above offers suggestions for all interested parties including the drug regulatory authorities, governments and the EU to readdress commitments to patients not only for ethical reasons but also for safeguarding patients’ treatment rights and safety. In this context, there is a need to establish of a central EU court, which in conjunction with the EMA and Europol can monitor unethical/illegal activity in the pharmaceutical industry and bring legal action against the relevant companies, which is a more appropriate and possibly more effective procedure than relying on individual member states.

## Figures and Tables

**Table 1:** The mode of action and clinical effects of the iron chelating drugs.

**Optimal chelation therapy for the normalisation of the iron stores in thalassaemia** The ICOC combination dose protocol of L1 (75–100 mg/kg/day) /DF (40–60 mg/kg/day, 3–7 days per week).
**Recommended dose range for the chelating drugs in thalassaemia major patients** Oral DFRA 20–40 mg/kg/day. Oral L1 75–100 mg/kg/day. Subcutaneous DF 40–60 mg/kg/day.
**Differential iron removal from various organs of iron loaded patients** DFRA preferential iron removal from the liver and L1 from the heart. DF from the liver or heart. (Efficacy in iron removal is related to the dose of all chelators).
**Efficacy in iron removal from the heart of iron loaded patients** The ICOC oral L1/intravenous DF combination > The ICOC oral L1/subcutaneous DF combination > oral L1> intravenous DF > subcutaneous DF > DFRA. (Efficacy in iron removal is related to the dose of the chelators).
**Route of elimination of increased iron excretion in iron loaded patients** DFRA: Faecal iron. L1: Urinary iron. DF: Mostly urinary but also faecal iron.
**Compliance of iron loaded patients with chelating drugs** Better compliance with oral DFRA and oral L1 in comparison to subcutaneous DF.
**Effect of chelating drugs on iron absorption** Increase of iron absorption by DFRA and other lipophilic chelators such as maltol and 8-hydroxyquinoline. Inhibition of iron absorption by the hydrophilic chelators DF and L1.
**Iron removal from diferric transferrin and NTBI in iron loaded patients** Effective transferrin iron removal only by L1 (estimated 40% iron removal from diferric transferrin at L1 concentrations > 0.1 mM), but not by DF or DFRA. All three chelating drugs are effective in the removal of non-transferrin bound iron (NTBI).
**Iron redistribution in diseases of iron metabolism by chelating drugs** L1 and to a lesser extent DF can cause iron redistribution from the reticuloendothelial system to the erythron in anaemic rheumatoid arthritis patients. Enterohepatic circulation by DFRA and metabolites.
**Increase in excretion or absorption of metals other than iron, e.g., Zn and Al** Order of increased Zn excretion in iron loaded patients: L1> DF > DFRA. DF and L1 cause increase Al excretion in renal dialysis patients. DFRA causes an increase in Al absorption.
**Iron mobilisation and excretion of chelator metabolite iron complexes** Several DF metabolites have iron chelation potential and increase iron excretion but not the L1 and DFRA glucuronide conjugate metabolites.
**Combination chelation therapy** DFRA, L1, DF and combinations are more effective in iron excretion than monotherapies. The L1/DF combination has been used effectively for more than 20 years. Different 1–3 chelating drug combinations are under evaluation.
**Chelating drug synergism with ascorbic acid (vitamin C)** Ascorbic acid acts synergistically with DF but not L1 or DFRA for increasing iron excretion. L1 inhibits the pro-oxidant effects of ascorbic acid.
**Antioxidant effects by chelating drug** L1 and DF have shown antioxidant action in in vitro, in vivo and clinical settings. The antioxidant effects of DFRA are under evaluation. Only L1 has been shown to have antioxidant effects in the brain of patients with Friedreich’s ataxia and pantothenate kinase-associated neurodegeneration.

Abbreviations: Deferasirox (DFRA). Deferroxamine (DF). Deferiprone (L1). International Committee on Chelation (ICOC). Non-transferrin bound iron (NTBI).

**Table 2:** Examples of the serious toxic side effects of deferasirox.

**Nephrotoxic effects** Renal function deterioration and damage leading to renal failure requiring temporary or permanent haemodialysis. Renal tubulopathy in young thalassaemia patients with low serum ferritin levels (<1.5 mg/L).
**Gastrointestinal effects** Fatal gastrointestinal haemorrhages, with higher frequency in elderly patients especially those with advanced haematologic malignancies and/or low platelet counts. Gastric ulcers, duodenal ulcers and esophagitis. Gastric intolerance (28–31%) and nausea.
**Haematological effects** Pancytopenia or aggravation of pancytopenia and thrombocytopenia. Patients with pre-existing haematological disorders that are frequently associated with bone marrow failure are mostly affected. Agranulocytosis in thalassaemia and other patients.
**Hepatic effects** Hepatic failure.
**Skin effects** Alopecia and erythema multiforme. Skin and subcutaneous tissue disorders.
**Metal metabolism and toxicity effects** Long-term use of deferasirox is suspected to cause an increase in toxic metal dietary absorption, e.g., Al and Fe which may lead to neurodegenerative diseases and also, e.g., Ni and Cd, which may lead to carcinogenesis
**Other toxic side effects** Fanconi syndrome Hyperchloremic metabolic acidosis Leukocytoclastic vasculitis Auditory and ocular toxicities Anaphylactic reactions Infections (39%). Further investigations are needed to prove the extent of implication of deferasirox, its metabolites and iron or other metal complexes

**Table 3:** The uses of iron chelating drugs in iron overload subgroups and prospects for use in other clinical conditions.

**Transfusional and non transfusional iron overloading diseases** **Haemoglobinopathies** β-Thalassaemia major, β-thalassaemia intermedia including non-transfusion-dependent-thalassaemia, HbE β-thalassaemia, HbS β-thalassaemia, sickle cell anaemia.
**Anaemias** Myelodysplasia, aplastic anaemia, sideroblastic anaemia, Blackfan–Diamond anaemia, Fanconis anaemia, pernicious anaemias, congenital dyserythropoietic anaemia, hereditary hypochromic anaemia, post-allogeneic hematopoietic stem cell transplantation
**Hereditary conditions** Idiopathic haemochromatosis, hereditary spherocytosis, pyruvate-kinase deficiency, congenital atransferrinaemia, porphyria cutanea tarda
**Post-allogeneic haematopoietic stem cell transplantation** Myelodysplasia, thalassaemia, sickle cell disease
**Iatrogenic** Intramuscular iron dextran, dietary or iatrogenic iron intake, iron poisoning
**Other iron loading conditions** Haemolytic disease of the newborn, iron overload in liver disease, iron overload in haemodialysis
**Iron imbalance and oxidative stress** Neurodegenerative diseases including Friedreich’s ataxia, Pantothenate kinase-associated neurodegeneration, Hallevorden–Spatz syndrome, Parkinson’s disease, Alzheimer’s disease Cyclooxygenase and lipoxygenase inhibitors Congestive cardiac failure, liver disease, acute kidney disease, rheumatoid arthritis Ischaemia reperfusion injury Drug toxicity, e.g., doxorubicin induced cardiac damage
**Iron imbalance in chronic diseases and iron deficiency** Anaemia of chronic disease in inflammatory, infectious and neoplasmic diseases. Iron deficiency anaemia.
**Free radical pathology** Cardiac, liver, kidney, neurological and all other diseases affected by free radical damage and oxidative stress leading to tissue damage. Ageing.
**Toxicity of environmental, diagnostic, therapeutic metals** Aluminium overload. Actinide contamination toxicity, e.g., plutonium, americium and uranium Diagnostic metal complexes toxicity, e.g., gallium, indium, gadolinium Therapeutic metal complexes toxicity, e.g., gold, platinum
**Other metal imbalance and toxicity conditions** Inhibition of all cancer types with increased iron requirements, neoplasmic disease, neuroblastoma, hepatocellular carcinoma. (Adjuvant therapies with anticancer drugs).
**Infectious diseases** Antimicrobial effects in microbial infections, e.g., meningitis, malaria and other parasitic infections, mucormycosis. (Adjuvant therapies with antimicrobial drugs).

## Data Availability

Not applicable.
